# A methodological study of virtual self-touch: effects on perception and motor control strategies

**DOI:** 10.3389/fbioe.2026.1819228

**Published:** 2026-06-22

**Authors:** Kotaro Okada, Kazuhiro Matsui, Ruu Fujii, Ryoma Kojima, Keita Atsuumi, Hiroaki Hirai, Atsushi Nishikawa

**Affiliations:** 1 Graduate School of Engineering Science, the University of Osaka, Toyonaka, Japan; 2 Graduate School of Information Science and Arts, Osaka Electro-Communication University, Shijonawate, Japan; 3 Graduate School of Information Sciences, Hiroshima City University, Hiroshima, Japan

**Keywords:** body schema, haptic, motor control, rehabilitation, virtual self-touch

## Abstract

**Introduction:**

Advances in virtual reality technology have facilitated research that leverages the plasticity of body schema. This study investigates whether the use of an avatar habituation method, in which participants voluntarily touch an avatar as a substitute body and receive haptic feedback, is crucial for updating body schema. This approach is called virtual self-touch (VST).

**Methods:**

We conducted an experiment with 10 participants using avatars that were the same size as the participants’ actual bodies and with the length of the right forearm increased by 1.5 times. The effectiveness of VST was evaluated using questionnaire-based sense of body ownership and agency, proprioceptive drift measurements, and reaching tasks without displaying the avatar. Further, the proposed method was compared with an existing avatar habituation method, the arm swing task (AST).

**Results:**

Our results demonstrated that VST produced larger changes in proprioceptive drift than AST. Furthermore, larger variations in the fingertip trajectories and reach endpoints were observed for VST than for the AST during the reaching task, which indicated that VST affects motor planning.

**Discussion:**

This suggests that VST contributes to changes in the body schema. Additionally, distortions in spatial perception were detected in the environment when only somatosensory feedback was relied on. This result provides insights about the representation of spatial perception in the body schema.

## 1 Introduction

Recently, research on motor control strategies using virtual reality (VR) for applications such as rehabilitation has been actively conducted (Sveistrup, 2004; Henderson et al., 2007; Levin et al., 2015; Kim et al., 2020). In response to this trend, we have proposed the concept of the Physio Avatar, which aims to induce physiological change through experience (Ando et al., 2024). We have shown that human motor control strategies change when a participant experiences avatars with different kinetic behavior from themself, and that after the participant experiences the avatar, the aftereffects of the error learning remain (Ando et al., 2023). In other words, after experiencing an avatar that has a different body, we can intervene in participant’s motor control strategies. We considered applying this not only to dynamics but also to the recognition of body size, that is, intervention in the body schema.

Humans utilize their bodies to perceive and interact with their environment, with their minds mediating this interaction. Additionally, humans employ tools to access their environment. By contrast, enabling a robot to use even a simple tool such as a pointing stick generally requires explicit modeling of the tool geometry and its functional endpoint, as well as motion planning adapted to that specific configuration. When the tool geometry changes, these models and plans often need to be redefined. Nevertheless, previous studies have demonstrated that humans can manipulate various tools as if they are extensions of their bodies by expanding peripersonal space and updating action-related body representations (Canzoneri et al., 2013; Maravita and Iriki, 2004). This theoretical framework for recognizing one’s body is referred to as the body schema, a concept proposed by Head and Holmes that represents a dynamic, unconscious process that governs body posture and movement (Head and Holmes, 1911), and has been subsequently validated in the domains of human motor control (Jacobs et al., 2009) and neuroscience (Maravita and Iriki, 2004).

The body schema (Head and Holmes, 1911) is plastic, and can be updated *via* multisensory experience (Iriki et al., 1996; Maravita et al., 2003). VR technology, which enables precise separation of visual information from other sensory modalities, is a powerful tool for studying body schema change (Spanlang et al., 2014; Bohil et al., 2011). It has demonstrated applications in rehabilitation (Imaizumi et al., 2016) and robot control (Lipton et al., 2018). To investigate this phenomenon, we focused on the forearm as a relatively simple and controllable body segment for evaluating body schema updates.

The rubber hand illusion (RHI) (Botvinick and Cohen, 1998) is a representative visuotactile illusion that modulates body ownership and image. At the same time, RHI-related proprioceptive drift has been employed as an indirect indicator of alterations in action-related body representations. The self-touch illusion (STI) is a development of the RHI (Ehrsson et al., 2005; Davies et al., 2013). Here, a blindfolded participant touches another person’s hand using their real hand while this other person touches another part of the subject’s body using their hand, causing the participant to perceive the other person’s hand as their own hand. They demonstrate that synchronous multisensory stimulation can transfer body ownership to a substitute limb and update body representations. Due to the inherent occlusion of the participant’s hands in HMD-based VR, the use of a blindfold is unnecessary. Furthermore, the synchronized tactile feedback can directly reinforce the sense of ownership (Gonzalez-Franco et al., 2022). This study proposes a novel approach, termed virtual self-touch (VST), a VR-based method in which participants voluntarily touch their avatar’s fingers with their own fingers while receiving synchronized haptic feedback. This method combines visual, tactile, proprioceptive, and agency cues to promote robust body schema updates.

We hypothesized that the presence of a size-altered avatar during VST would induce changes in proprioceptive drift and reaching task performance relative to the same-size avatar (SA) condition, in which the avatar matched each participant’s body size. We further predicted that these effects would differ quantitatively from those observed with the Arm Swing Task (AST), an avatar habituation task in which participants repeatedly swung their arms to become accustomed to each avatar’s size.

## 2 Related works

### 2.1 Our previous works

Our previous study introduced a size-adjustable avatar system that matches the user’s body dimensions in a standalone head-mounted display environment. In this study, we present a comprehensive experimental report, encompassing detailed protocols, evaluation-task outcomes, and a discussion centered on motor control strategies.

### 2.2 Body schema and its manifestation

The definition of body schema has been the subject of extensive debate, and a fully unified definition remains elusive (Poeck and Orgass, 1971; de Vignemont et al., 2021; Sattin et al., 2023). In accordance with the foundational distinction initially introduced by Head and Holmes (Head and Holmes, 1911), the term “body schema” is employed to represent a dynamic, unconscious process that governs body posture and movement. This schema can be conceptualized as a bottom-up, action-oriented representation that guides motor commands for planning and online movement control (Hansford et al., 2024; Pitron et al., 2018; Haans and IJsselsteijn, 2012; Preston and Newport, 2011). The term “body image” is employed to denote the conscious representation one holds with regard to one’s own body. This concept encompasses perceptual and conceptual dimensions of bodily self-perception (Haans and IJsselsteijn, 2012; Preston and Newport, 2011). Therefore, multisensory integration is imperative for the functionality and learning of the body schema (Iwamura, 1998).

The body schema is not a numerical model or organ; rather, it is a conceptual framework. However, the existence of the body schema manifests as various observable phenomena, which then serve as external evidence of its existence. In addition, the concept of peripersonal space (PPS) is a manifestation of the body schema in spatial cognition (Làdavas and Serino, 2008). PPS is “a multisensory representation of the space immediately surrounding the body that uses one or multiple body parts as spatial references to encode nearby objects” (Patané et al., 2019). Moreover, multisensory integration has confirmed that the spatial perception characteristics in the PPS differ from those in other spaces. These change with the use of tools, for example, and affect motor planning (Iriki et al., 1996). Changes in the body schema can be captured indirectly by detecting changes in its manifestations.

### 2.3 Self-touch illusion

The concept of the STI originated from Ehrsson et al., who tested the somatosensory role of the RHI to examine whether the relationship between the RHI and brain activity could be influenced by observing one’s hand (Ehrsson et al., 2005). In Ehrsson et al.’s experiment, the operator moved the participant’s left index finger (with the participant’s eyes closed) to touch the joint of a rubber hand’s right index finger while the participant simultaneously touched the operator’s right index finger. These touch movements were repeated synchronously at a frequency of 1 Hz, inducing an effect similar to that of the RHI. Davies et al. experimented on the STI and RHI, comparing specific conditions to verify the effects of such conditions on the said illusions (Davies et al., 2013). Unlike Ehrsson et al.’s experiment, Davies et al.’ experiment involved using a paintbrush to stroke corresponding areas on the left and right hands to compare the RHI and STI. Davies et al. reported that STI effects diminished but did not disappear when the right and left hands were separated or angled differently, whereas the RHI was relatively unaffected by these conditions.

Pilacinski et al. established these experimental setups in VR environments and reported the phantom touch illusion (PTI), where a tingling sensation was induced by performing self-touch without synchronized tactile stimulation (Pilacinski et al., 2023). Like Davies et al.’ setup, Pilacinski et al.’s experiment involved self-touch using tools. However, this can be considered a variant of the RHI, and unlike both the RHI and STI, the PTI induced ownership-related responses based on somatosensory and visual information without using tactile sensations.

Ehrsson et al. suggested that synchronously tapping corresponding areas could induce the sense of body ownership over a rubber hand, Davies et al. highlighted the continued importance of visual information for body ownership, and Pilacinski et al. indicated that these effects could also be induced in VR environments. Building on these studies, the present research aims to promote a sense of body ownership and agency over the avatar through VST using synchronized touch and visual recognition within VR environments.

### 2.4 Evaluation of avatar-induced body representation

No standardized metric has been developed to evaluate the extent to which humans perceive nonbody objects as part of their bodies. Therefore, the present study utilizes complementary indices, including questionnaire-based sense of body ownership and agency, proprioceptive drift as an implicit body-representation index (Inamura et al., 2017), and a reaching task to capture body schema-related changes in motor planning (Umezawa et al., 2022).

Inamura et al. experimentally tested whether the subjective length of an arm would change when a participant experienced avatars of different sizes in a VR environment. Their experiment assessed avatar-induced body-representation changes in the forearm by having participants experience avatars at 0.5x, 1x, and 2x scales. After the habituation task, the participants removed the head-mounted display (HMD), and their proprioceptive drift was measured by having them evaluate the length of their right arm using a scale-covered shield on their right arm. Proprioceptive drift also occurs in RHI experiments (Gallagher et al., 2021), and this drift is independent of self-reported ownership (Shibuya et al., 2021). In the present study, the method is regarded as an implicit index of body-representation change and is interpreted in conjunction with questionnaire-based ownership/agency results.

Umezawa et al. experimentally examined changes in the body schema caused by the ownership of an independent prosthesis, specifically by developing a “sixth finger” and evaluating motor planning and perception changes caused by ownership of it. One of the evaluation methods used in this study was a reaching task where a participant avoided obstacles while attempting to reach a target line positioned far from a starting point. The authors detected changes in the body schema by evaluating the area the participant reached in relation to the target line while obstacles impeded the shortest path. Umezawa et al. expected that ownership of the sixth finger would lead to a perception of a larger hand, increasing its clearance from obstacles. The body schema affects not only simple spatial perception but also motor planning. For studies on its influence on motor planning, the reaching task on a desktop plane is an appropriate evaluation method due to its low degree of freedom. In this study, the reaching task from Umezawa et al.’s experiment is modified by using cylindrical obstacles to evaluate not only the reached endpoint but also the clearance along the path, indicating changes in the body schema.

## 3 Method and experiment

The study was a within-participants, 2-day proof-of-concept that compared two avatar habituation methods under two avatar conditions. On each day, participants completed a pretest evaluation, an avatar habituation task, and post-test evaluations under a same-size avatar (SA) and an enlarged avatar (EA). On day 1, the arm swing task (AST) was employed as the habituation method, while on day 2, the proposed virtual self-touch (VST) was utilized. The primary outcome measures included the following: (i) questionnaire-based assessments of body ownership (SoO) and agency (SoA), (ii) proprioceptive drift as an implicit index of body-representation change, and (iii) a reaching task to capture changes in motor planning.

### 3.1 Objectivities and overview

In this study, we propose VST, where a participant uses an avatar’s finger to touch another finger of the avatar, with haptic feedback provided by a vibrator, as a means of inducing a sense of body ownership and agency over avatars of different sizes. We verified the effectiveness of VST by comparing it with a previously reported avatar habituation method that uses arm-swinging motions (Inamura et al., 2017).

We targeted the right forearm as the body part for intervention because this was the same area tested by Inamura et al. In the experimental environment, the actual lengths of the participants’ upper limb segments were measured, and avatars corresponding to these measurements were used. We investigated the ownership/agency effects on two types of avatars: one with the same forearm length as the participant (hereinafter called same-size avatar [SA]) and one with a longer (by 1.5 times) forearm (hereinafter called enlarged avatar [EA]). If the forearms of the avatar are shortened, the participant may accidently touch other parts of their real body. However, if the forearms of the avatar are too long, the participant may not be able to touch the avatar. Therefore, we chose an extension of the avatar arm length of 1.5-times for the pilot study, following Nakamura’s study (Nakamura et al., 2014).

The experiment consisted of three phases: a habituation task, an evaluation task, and a questionnaire. The habituation task was designed to help the participants adapt to the avatars through specific movements, the evaluation task aimed to assess the participants’ body perception, and the questionnaire was used to investigate the participants’ sense of agency and ownership of their avatars’ bodies. The experiment was conducted in the following order. Inamura et al. assumed that the avatar-related sense of body ownership and agency would be overwritten with each new condition, so the SA and EA conditions were not randomized here. The name of each phase is enclosed in parentheses.

1. Evaluation task (pretest)
2. Questionnaire (SA pre-questionnaire)
3. Habituation task (SA habituation)
4. Questionnaire (SA post-questionnaire)
5. Evaluation task (SA test)
6. Switch from SA to EA
7. Questionnaire (EA pre-questionnaire)
8. Habituation task (EA habituation)
9. Questionnaire (EA post-questionnaire)
10. Evaluation task (EA test)

Each participant wore an HMD at all times during the experiment. The visibility of the avatar could be toggled on and off, depending on the task requirements.

The experiment was conducted over 2 days, and the two types of habituation tasks were compared. On the first day, the habituation task involved arm swinging; on the second day, it involved self-touch. Due to scheduling constraints, not all participants completed the experiments on consecutive days. However, for each participant, the two sessions were separated by at least 24 h. This interval was introduced as a practical washout period to mitigate potential carryover effects while maintaining a fixed task order across participants. This design was based on prior studies reporting that body ownership can be induced within minutes (Kokkinara and Slater, 2014; Slater et al., 2008) and can diminish shortly after stimulation ends (Finotti et al., 2023). Furthermore, during each session, participants were instructed to maintain the HMD for as long as possible to minimize unintended disruption of the induced effects.

A total of 11 healthy adults participated in this study. However, one participant discontinued due to VR sickness, leaving 10 valid participants (eight males and two females). This study was designed as a methodological proof-of-concept. The sample size was set to 10, which is comparable to those used in related studies (Nakamura et al., 2014; Botvinick and Cohen, 1998; Tan et al., 2024) and is consistent with the small sample sizes commonly employed in body representation and motor control studies (Shadmehr and Mussa-Ivaldi, 1994). These precedents suggest that sample sizes of approximately 8–10 participants are commonly used in experimental studies in this domain. The present study adopted a within-participant design, in which each participant experienced all experimental conditions. This design reduces interindividual variability and increases statistical sensitivity, allowing for the reliable detection of condition-dependent changes, even with a relatively small sample size. However, the relatively small sample size may limit the generalizability of the findings, and further studies with larger cohorts are necessary. The average age of the participants was 23.6±1.7 years. All participants were right-handed, as determined *via* self-report. They received an oral explanation of the study’s purpose and methods and consented to participate. Additionally, the experiment was conducted with the approval of the Ethics Committee for Research Involving Human Subjects at Graduate School of Engineering Science, the University of Osaka (R3-17-2).

### 3.2 Haptic feedback device

**FIGURE 1:**
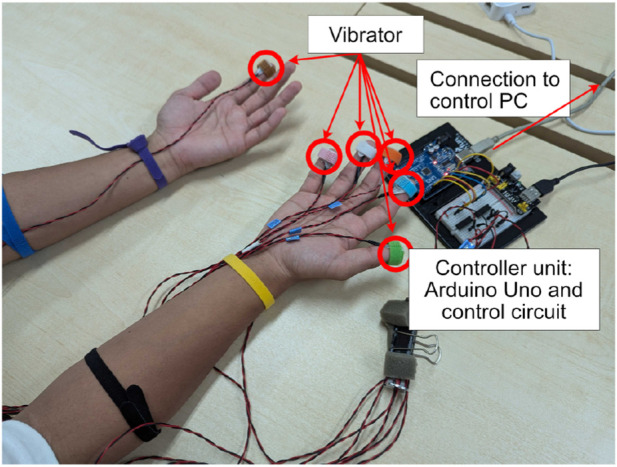
Haptic feedback device.

In the self-touch task, feedback upon fingertip contact was provided using not only visual information but also haptic information. This was achieved virtually by developing a haptic feedback device that delivers vibratory stimuli to the fingertips. The appearance of the device is shown in Figure 1.

The haptic feedback device consisted of a controller unit and a vibration unit. The controller unit was composed of a microcontroller and a control circuit. The controller received signals from a personal computer (PC) and controlled the ON/OFF status of each vibrator. The vibration unit contained six eccentric-motor vibrators, which were attached to the distal phalanx of the left index finger and the distal phalanges of the fingers on the right hand. Additional hardware specifications can be found in the Supplementary Material.

### 3.3 Experimental environment and equipment

**FIGURE 2:**
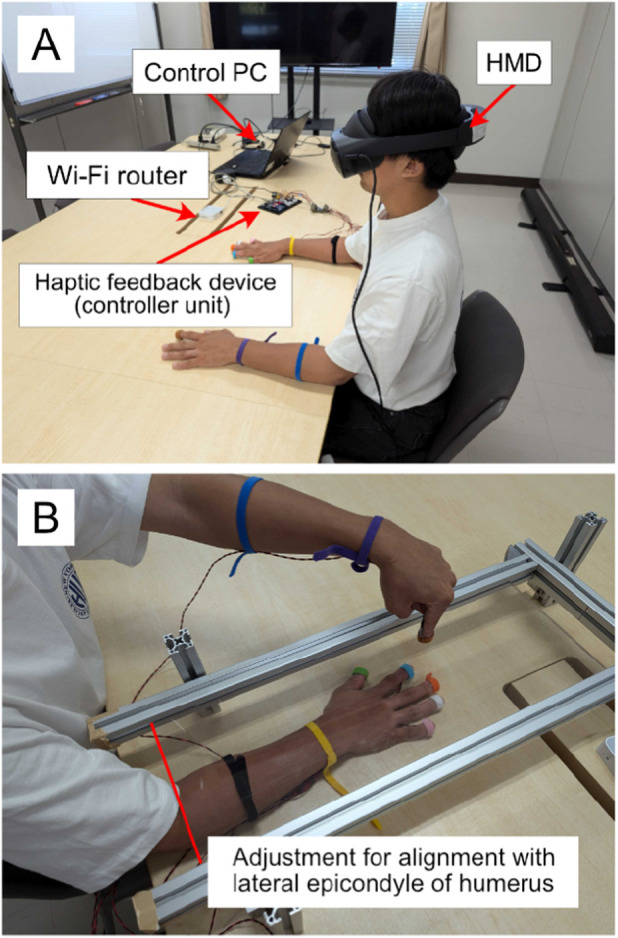
**(A)** Experimental environment, **(B)** measuring instruments for forearm length.

The experiment was conducted in an indoor laboratory of the affiliated institution. The experimental system consisted of an HMD, a control PC, the haptic feedback device, and a Wi-Fi router. Additionally, during the evaluation task, measuring instruments for forearm length were used, as shown in Figure 2.

The HMD employed in the study was the Meta Quest Pro (Meta), which utilizes built-in sensors for upper-limb tracking, facilitating avatar control. The VR application was developed using the Unity game engine (Unity Technologies) with the Meta XR Interaction Software Development Kit (Meta, 2024). The Meta Quest Pro is a standalone HMD, i.e., the VR application ran directly on the HMD. This results in a display delay for the avatar of approximately 20 ms. A control application on the control PC was responsible for facilitating communication with the HMD *via* Wi-Fi, acquiring logs, and transmitting signals to the haptic feedback device. Comprehensive software versions and network configurations are provided in Supplementary Material.

The movement data of the user, obtained from the built-in sensors of the HMD, were logged into the control PC *via* communication. These logs were sampled at a rate of 50 Hz.

When the contact detection areas on the avatar’s fingertips touched each other, the VR application sent information to the control application about which fingers made contact. The control application then transmitted ON signals corresponding to the touched fingers to the haptic feedback device, which was connected to the control PC *via* USB. The ON/OFF status of each vibrator was controlled *via* serial communication.

### 3.4 Avatar

**FIGURE 3:**
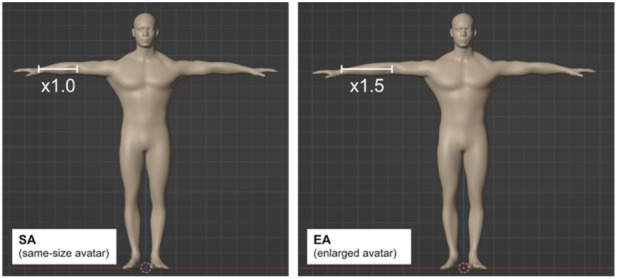
Visual of avatars (left: SA, right: EA).

The avatars used in the experiment were created to match the participants’ body sizes (Figure 3). The base mesh for the avatars was derived from Blender’s Human Base Meshes (version 1.1) (Blender Foundation, 2024). A system was developed to deform this mesh according to each participant’s body measurements. Mesh deformation was achieved by specifying the lengths of the embedded rigs and then scaling the mesh along the rig axis accordingly.

The avatar sizes were determined by obtaining the necessary body length measurements before the experiment. These measurements were the lengths of the upper arm, forearm, and index finger on the left and right sides. The upper-arm length was measured from the acromion to the lateral epicondyle of the humerus; the forearm length, from the lateral epicondyle of the humerus to the ulnar styloid process; and the finger length, from the ulnar styloid process to the tip of the index finger. The SA and EA were created using these measurements.

The avatars were generated using the Blender software and exported in VRM format (VRM Consortium, 2024). Each VRM file was loaded into the VR application, allowing different avatars to be displayed, depending on the participant and avatar conditions.

### 3.5 Basic posture

In this experiment, each participant sat in a chair and placed their forearms on a table placed in front of them. The table was a standard office desk without any anti-slip coating. Each participant was instructed to adopt the following basic posture:

- Sit with the trunk perpendicular to the table. Participants had to be positioned such that the sagittal plane forms an approximately 90-degree angle with the chair seat.
- Ensure that the forearms contact the table.
- Maintain an elbow joint angle of approximately 120°, defined by the segments connecting the wrists and elbows and those connecting the elbows and shoulders.
- Maintain a shoulder joint abduction angle of roughly 10°.
- Keep the fingers slightly spread, ensuring the palms contact the table.

The forearm posture was the angle formed by the line connecting the wrist and elbow, and the frontal plane of the trunk, with the clockwise direction being positive; this was called the forearm angle. Thus, extending the arm straight forward from the body indicated a forearm angle of 90°. The forearm posture varied according to each task. Resetting to the basic posture was generally conducted by the experiment operator, who moved the participant’s arm, and participants were instructed not to move actively unless directed otherwise.

### 3.6 Habituation task

The tasks designed to help the participants adapt to the avatars were called habituation tasks. In this study, we conducted two types of these activities: the arm swing task (AST) and the VST. These tasks were performed with the avatar displayed.

#### 3.6.1 Arm swing task (AST)

**FIGURE 4:**
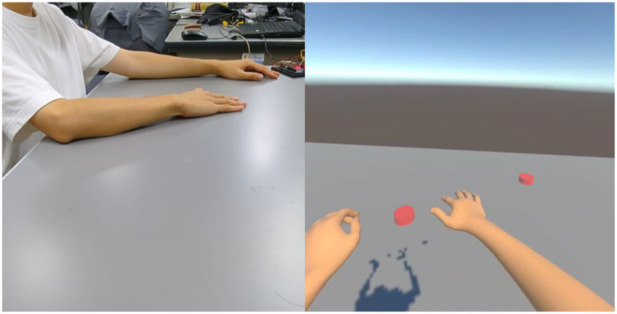
Participants performing the AST task. Left: real space; right: virtual space.

The AST is the habituation method adopted by Inamura et al. This task involves controlling the avatar through the flexion and extension of the participant’s elbow joint and observing these movements as shown in Figure 4. This method links the somatosensory and visual information of the avatar arm length, promoting the ownership/agency and body-schema-related effects over the avatar. The following protocol was used for the AST in this experiment:

1. The operator positioned the participant in the basic posture.
2. In the virtual space, a semitransparent red cylinder with a diameter of 5 cm and a height of 1 cm was displayed, centered at the avatar’s right index fingertip. An identical cylinder was displayed at a location 30° counterclockwise from a cylinder centered at the elbow.
3. A metronome sound was played at 2 s intervals using the HMD’s built-in speakers.
4. The participant was instructed to move the avatar’s right index fingertip back and forth between the two cylinders in sync with the metronome sound. This movement was performed centered at the elbow, with the wrist and finger joints fixed. Additionally, the participant was instructed to keep the forearm and hand contacting the table during the movement.
5. The participant was instructed to focus on the avatar’s forearm and hand during the movement.
6. Upon the operator’s signal, the above movement was performed for 5 min. The operator measured the time and gave signals at the 150 s mark and at the end of the 5 min period. The participant stopped moving upon the operator’s signal at the 5 min mark, and the operator repositioned the participant in the basic posture.

#### 3.6.2 Virtual self-touch (VST)

**FIGURE 5:**
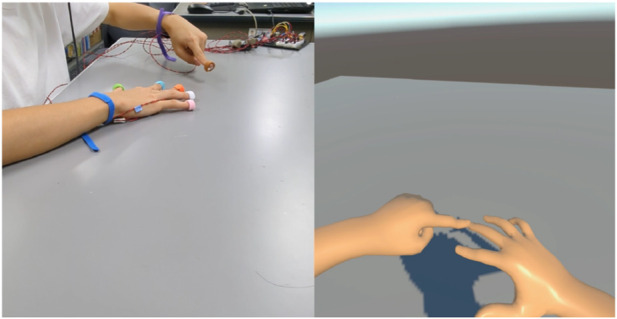
Participants performing the VST task. Left: real space; right: virtual space.

VST is the proposed habituation method. The VST involves using a haptic feedback device to touch an avatar’s fingers using their own fingers as shown in Figure 5. This triggers haptic feedback through vibrators, thereby promoting the ownership/agency and body-schema-related effects over the avatar. Synchronous visuotactile stimulation, including vibrotactile feedback, has been demonstrated to induce a sense of body ownership (D’Alonzo and Cipriani, 2012; Kokkinara and Slater, 2014) The following protocol was used for the VST:

1. The operator first positioned the participant in the basic posture, then moved their arm such that the right forearm was approximately at 30°. This angle was chosen because it allowed participants to comfortably perform the self-touch task without physical strain and reach all fingertips of the right hand with the left index finger.
2. The participant was instructed to keep the right-hand fingers sufficiently spread and keep the palm in contact with the table.
3. A metronome sound was played at 2 s intervals using the HMD’s built-in speakers.
4. The haptic feedback device was activated. This caused the vibrators in the distal phalanges to vibrate when the contact detection areas at the avatar fingertips made contact.
5. The participant was instructed to touch each finger of the avatar’s right hand sequentially with the avatar’s left index finger in time with the metronome. The sequence started from the thumb and proceeded to the little finger, and then from the little finger to the thumb. The participant was instructed to touch the fingertips of the avatar, even if they touched the physical body. If they could not accurately touch the avatar’s fingertips within 2 s, then they were asked to move to the next finger in time with the metronome.
6. The participant was instructed to focus on the avatar’s forearm and hand during the movement.
7. Upon the operator’s signal, the above movement was performed for 5 min. The operator measured the time and gave signals at the 150 s mark and at the end of the 5 min period. The participant stopped the task upon the operator’s signal at the 5 min mark, and the operator repositioned the participant in the basic posture.

### 3.7 Questionnaire

Refer to previous research of motor control (Nagai et al., 2024; Okamoto et al., 2024; Osumi et al., 2018; Katayama et al., 2018), a questionnaire was administered orally to obtain the participants’ subjective evaluation of their sense of body ownership and sense of agency over the avatars. These indices, sense of body ownership and agency, are typical indices in motor control (Braun et al., 2018). The questionnaire was administered before and after the habituation tasks, and minor avatar control (such as arm swings and hand grips) was allowed during questionnaire completion. However, actions equivalent to self-touching were prohibited, such as touching the right hand with the left hand. Therefore, the questionnaire was administered orally to minimize interruption of the VR experience, e.g., avoiding HMD removal. Despite the potential for response bias, the participants were instructed to respond freely and without evaluation. This potential bias is acknowledged as a methodological limitation.

In the questionnaire, participants responded using seven-point Likert scales to indicate their sense of body ownership and sense of agency. In particular, when participants experience an avatar that differs from their own body, the sense of body ownership and sense of agency can be regarded as key subjective measures of how participants relate the avatar to their own body (Tsakiris et al., 2006; D’Angelo et al., 2018). The participants rated their sense of body ownership from 1 (I did not feel at all as if the avatar were my body) to 7 (I completely felt as if the avatar were my body). They rated their sense of agency from 1 (I did not feel at all as if I were controlling the avatar) to 7 (I completely felt as if I were controlling the avatar).

### 3.8 Evaluation tasks and metrics

The effectiveness of the avatar habituation methods was determined by conducting evaluation tasks before and after the habituation tasks. In this study, we conducted two types of evaluation tasks: the reaching task and proprioceptive drift measurement. Order effects were considered by having half of the participants perform the reaching task first while the other half measured their proprioceptive drift first. The avatar display was turned off during the evaluation tasks except during the initial position adjustments in the reaching task, capturing the participants’ body schema without relying on visual information.

#### 3.8.1 Measurement of proprioceptive drift

Proprioceptive drift is defined as a systematic displacement in the perceived position of one’s own limb toward a seen or imagined substitute body part (Botvinick and Cohen, 1998). When a participant encounters an avatar with an elongated forearm, this drift can manifest as a shift in the perceived position of the limb toward the avatar’s distal part, which may lead to an apparent elongation of the arm (Kilteni et al., 2012). Influenced by the method used by Inamura et al., we designed a procedure to measure proprioceptive drift: the participants were asked to point to the fingertip of one hand using the other hand while the avatar was not displayed. The protocol for proprioceptive-drift measurement was as follows:

1. The avatar display was turned off.
2. The operator positioned the participant in the basic posture.
3. A measuring device (Figure 2) was placed on the upper forearm of the participant. The end of the device’s top plate was adjusted to align with the lateral epicondyle of the humerus.
4. The participant was instructed to point to their right index finger using their left index finger upon a signal.
5. Upon the signal, the participant pointed to their right index finger. The operator measured the distance between the end of the device’s top plate and the tip of the left index finger.
6. The operator moved the participant’s left hand to return them to the basic posture.
7. Steps 5 and 6 were repeated a total of 10 times.

The length measured in this experiment represented the participant’s subjective distance from their right elbow to the right index fingertip. Given the variation in the actual body sizes of the participants, the collected data were normalized using the actual distance from the elbow to the right index fingertip. The normalized data were then used to evaluate proprioceptive drift comprehensively across all participants. In addition, for these normalized data, the pretest-normalized proprioceptive drift, in which the SA and EA tests were normalized by the proprioceptive drift of the pretest, was obtained for each participant to compare the difference between SA and EA.

### 3.8.2 Reaching task

The reaching task involves moving a fingertip to a target position while avoiding obstacles. In this study, we designed a reaching task where a participant navigates around cylindrical obstacles in a VR space, moving their fingertip from an area near their trunk to an area far from them. By having the participants perform the reaching task with the avatar turned off, we evaluated changes in the participants’ body schema based on their clearance from obstacles and the reached endpoint.

The reaching task was conducted according to the following protocol:.

**FIGURE 6:**
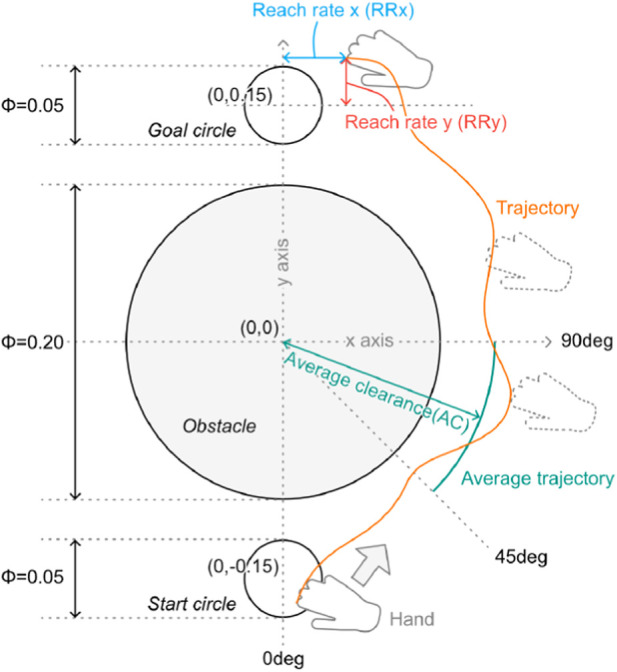
Outline of reaching task evaluation metrics and coordinate definition of evaluation space.

1. The operator positioned the participant in the basic posture. The avatar display was turned on in this stage due to a software specification (obstacles were generated based on the avatar position).
2. The operator moved the participant’s arm such that the forearm angle of the right arm was approximately 10°.
3. Obstacles, a start circle, and a goal circle were displayed in front of the participant, starting from their right index finger. The start circle was placed on the participant’s side of the obstacle, and the goal circle was placed on the opposite side. The obstacles were cylinders with a diameter of 20 cm and a height of 1 cm and were positioned between the start and goal circles. The specific coordinates are shown in Figure 6.
4. The participant was instructed to move their fingertip from the start circle to the goal circle without touching the obstacles, to reach the target position in the shortest, fastest way while keeping their palm in contact with the table during the movement.
5. The avatar display was turned off.
6. Upon hearing a “go” signal from the operator, the participant began attempting to reach the target.
7. When the participant subjectively felt they had reached the goal, they stopped the movement and said “ok.” The operator recorded the data based on the “ok” signal, marking it as the reach completion point.
8. The participant moved their arm back to the initial position. The operator displayed the avatar again and instructed the participant to position their fingertip back inside the start circle. The avatar display was turned off after repositioning.
9. Steps 6 to 8 were repeated a total of 20 times.

The evaluation used the sensor-acquired position data of the participant’s right index finger. These position data were preprocessed by transforming them into a planar coordinate system, with the center of the start circle set at the initial position and the center of the obstacle set at the origin. Although the data were acquired at a sampling rate of 50 Hz, the measurement duration varied between trials, so the data from each trial were interpolated and temporally normalized. The post-interpolation data-point number was set to 100, and cubic spline interpolation was used for interpolation. Using the interpolated data, we evaluated the participants’ clearance from obstacles and their accuracy in the reaching task. The representative values used for evaluation are shown in Figure 6.

A straight line connecting the origin and the center of the start circle was defined as the baseline, and the angle between the fingertip position line (connecting the fingertip position and the origin) and the baseline was defined as the fingertip position angle. For the fingertip–obstacle clearance, we evaluated data from the period when the fingertip position angle ranged between 45 and 90°, which was assumed to be from the beginning to the complete avoidance of the obstacle. The average clearance was calculated as a representative value called AC and used as the clearance representative value for each trial.

For the reaching distance, the distance between the fingertip position and the goal circle was evaluated. To separate the influence along each axis, we introduced two metrics: reaching rate x $\left(RR_{x}\right)$ and reaching rate y $\left(RR_{y}\right)$ . Reaching rate x $\left(RR_{x}\right)$ is the x-axis error between the fingertip and the center of the goal circle, and reaching rate y $\left(RR_{y}\right)$ is the y-axis error between the fingertip and the center of the goal circle. These metrics were influenced by the evaluation method used by Umezawa et al. Specifically, with the fingertip position coordinates denoted as (x,y) and the goal circle center coordinates denoted as $\left(x_{g},y_{g}\right)$ , $RR_{x}$ and $RR_{y}$ are defined in Equations 1, 2:

$$RR_{x}=x-x_{g}$$ (1)

$$RR_{y}=y-y_{g}$$ (2)

The average values of $RR_{x}$ and $RR_{y}$ over the last 10 time points of the recorded trajectory data, denoted as $\bar{RR_{x}}$ and $\bar{RR_{y}}$ , respectively, were used for evaluation.

Considering potential differences in environmental conditions, such as the presence or absence of vibrators, and possible variations in movement control strategies across different days, we performed a unified evaluation of the effects of the habituation tasks by normalizing the representative values of each trial based on the pretest values. This process, called pretest normalization, was implemented through the following steps, where the representative values refer to the AC , $\bar{RR_{x}}$ , and $\bar{RR_{y}}$ of each trial:

1. The representative values for each trial during the pretest were calculated for each participant.
2. The averages of the representative values across all trials of the pretest were computed for each participant, which served as the baseline values.
3. For the SA and EA tests, the baseline value was subtracted from the representative value of each trial to obtain the pretest-normalized value. The baseline value differed between participants.

Each trial’s representative values were assessed using the pretest-normalized values. Henceforth, “representative values” generally refers to the pretest-normalized values.

### 3.9 Statistical analysis

We used the Wilcoxon signed-rank test to examine the significant differences between each group (Wilcoxon, 1945). Due to the small sample size, normality could not be assumed, but the Wilcoxon signed-rank test (the nonparametric equivalent of the paired t-test) was applied because the sample sizes were the same across the compared groups. The significance level was set to 0.05, and Bonferroni correction was applied as needed to account for multiple comparisons.

In addition, effect sizes were calculated as needed for comparison. The effect size was calculated using r for the Wilcoxon signed-rank test. Computations were performed using the Equation 3 (Field, 2009), where z is the normal approximation statistic of the Wilcoxon signed-rank test and n is the sample size:

$$r=\frac{\left\|z\right\|}{\sqrt{n}}$$ (3)

## 4 Results

### 4.1 Questionnaire results

**FIGURE 7:**
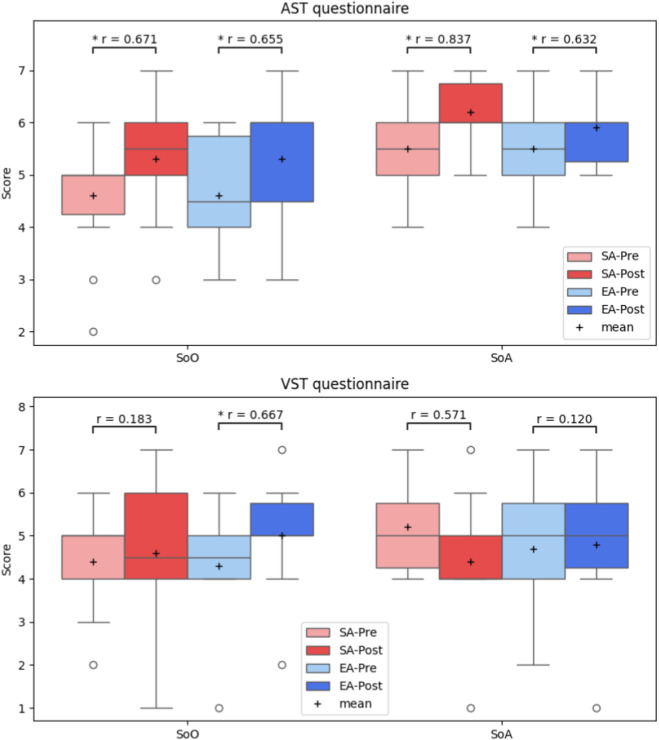
Questionnaire results. SoA: sense of agency, SoO: sense of body ownership. Top: AST questionnaire, bottom: VST questionnaire. r is effect size, and * means p-value <0.05 . n=10 .

The questionnaire results are shown in Figure 7. We identified any significant difference in the sense of agency and sense of body ownership before and after each habituation task for these questionnaires. The Wilcoxon signed-rank test was used for significance testing at a significance level of 0.05. The sample size was n=10 . In the AST, significant differences were observed in the sense of agency and body ownership before and after the SA and EA tests (SoA SA: z=−2.646 , r=0.837 , p=0.008 ; SoA EA: z=−2.000 , r=0.632 , p=0.046 ; SoO SA: z=−2.121 , r=0.671 , p=0.034 ; SoO EA: z=−2.070 , r=0.655 , p=0.038 ). In the VST, a significant difference was observed only in the SoO before and after the EA test ( z=−2.111 , r=0.667 , p=0.035 ); no significant differences were found under the other conditions. Thus, in the AST, both SoA and SoO showed significant pre-post differences under both avatar conditions, whereas in the VST, a significant pre-post difference was observed only for SoO under the EA condition.

### 4.2 Measurement of proprioceptive drift

**FIGURE 8:**
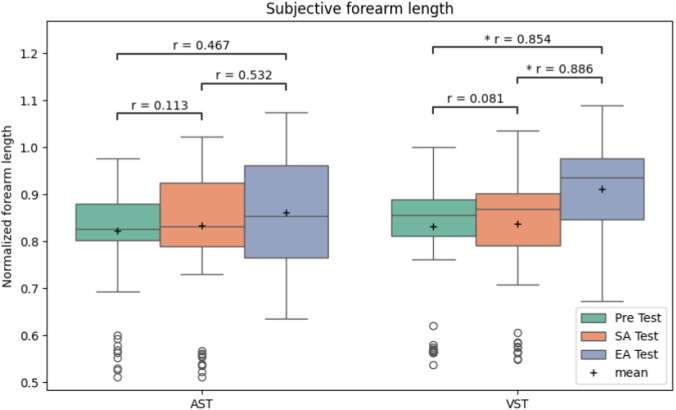
Results of proprioceptive-drift measurement. r is the effect size, and * means p-value <0.05 . n=10 .

The proprioceptive-drift measurement results are shown in Figure 8.

In the AST, the mean ± standard error of the perceived forearm length was 0.82±0.10 in the pretest, 0.83±0.12 in the SA test, and 0.86±0.12 in the EA test. The Wilcoxon signed-rank test was conducted for each group to investigate their significant differences in proprioceptive drift. The significance level was set to 0.05, and Bonferroni correction was applied for multiple comparisons, with the significance level adjusted to 0.05/3=0.0167 . No significant differences were observed between any pair of tests after the Bonferroni correction (Pre–SA: z=−0.357 , r=0.113 , $p_{\text{adj}}=1.000$ ; SA–EA: z=−1.682 , r=0.532 , $p_{\text{adj}}=0.278$ ; Pre–EA: z=−1.478 , r=0.467 , $p_{\text{adj}}=0.418$ ). The mean value was numerically highest in the EA test.

In the VST, the mean ± standard error of the perceived forearm length was 0.83±0.10 in the pretest, 0.84±0.11 in the SA test, and 0.91±0.10 in the EA test. The same Wilcoxon signed-rank test was conducted for each group as in the AST to identify significant differences in proprioceptive drift. Significant differences were observed between the SA test–EA test and pretest–EA test results (SA–EA: z=−2.803 , r=0.886 , $p_{\text{adj}}=0.013$ ; Pre–EA: z=−2.701 , r=0.854 , $p_{\text{adj}}=0.021$ ).

**FIGURE 9:**
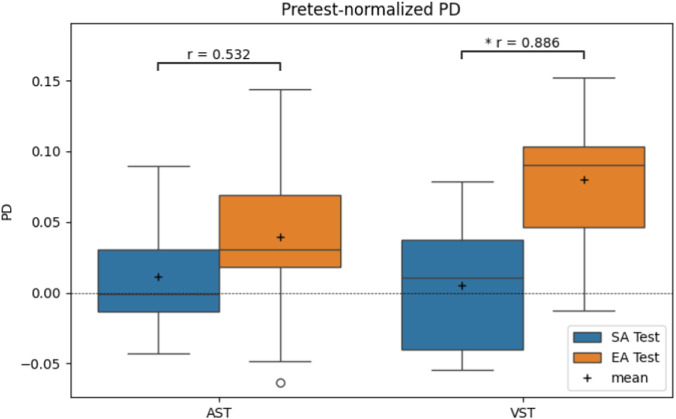
Results of pretest-normalized proprioceptive-drift (PD) measurement. r is the effect size, and * means p-value <0.05 . n=10 .

The pretest-normalized proprioceptive drift results are shown in Figure 9. The Wilcoxon signed-rank test was used for significance testing at a significance level of 0.05. The sample size was n=10 . A significant difference was observed in the VST between the SA and EA tests ( z=−2.803 , r=0.886 , p=0.005 ). However, in the AST, no significant difference was observed ( z=−1.682 , r=0.532 , p=0.093 ). Thus, a significant SA–EA difference in pretest-normalized proprioceptive drift was observed only in the VST.

### 4.3 Reaching task

We found after the experiment that the log data for the reaching task of one participant were lost due to system failure. Therefore, the reaching task was evaluated using only the data of the nine other participants (eight males and one female, with an average age ± SD of 23.6±1.8 years).

**FIGURE 10:**
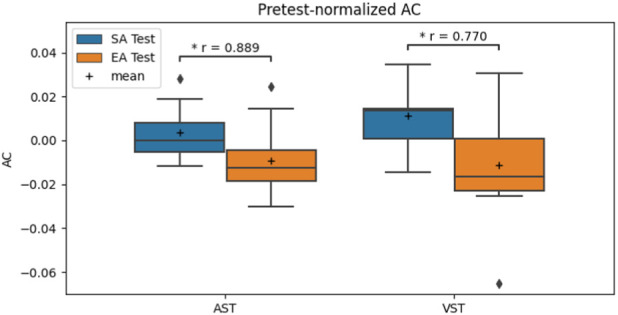
AC for AST and VST. r is the effect size, and * means p-value <0.05 . n=9 .

The AC results for the AST and VST are shown in Figure 10.

Here, the significance of the differences between the SA and EA tests under each condition of the AST and VST was examined using the Wilcoxon signed-rank test at a significance level of 0.05. Significant differences were observed between the SA and EA tests in both the AST ( z=−2.666 , r=0.889 , p=0.008 ) and the VST ( z=−2.310 , r=0.770 , p=0.021 ).

**FIGURE 11:**
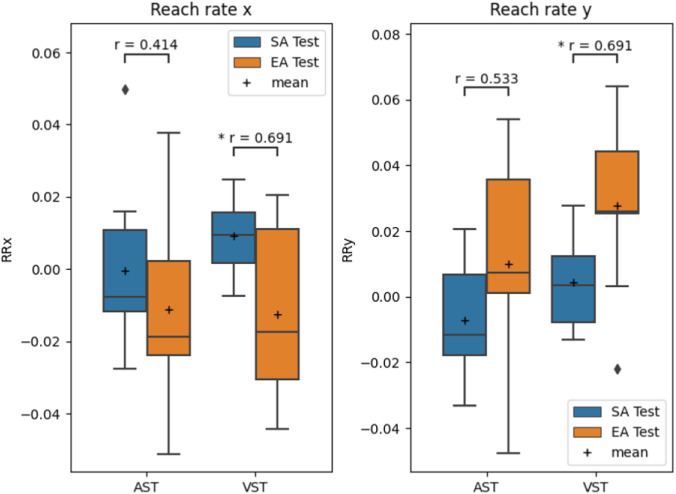
$\bar{RR_{x}}$ and $\bar{RR_{y}}$ for AST and VST. r is the effect size, and * means p-value <0.05 . n=9 .

Next, the $\bar{RR_{x}}$ and $\bar{RR_{y}}$ results for the AST and VST are shown in Figure 11.

Here, the significance of the differences between the SA and EA tests under each condition of the AST and VST was examined using the Wilcoxon signed-rank test at a significance level of 0.05. Only significant differences were observed in mean $\bar{RR_{x}}$ and $\bar{RR_{y}}$ in the VST between the SA and EA tests ( $\bar{RR_{x}}$ : z=−2.073 , r=0.691 , p=0.038 ; $\bar{RR_{y}}$ : z=−2.073 , r=0.691 , p=0.038 ). No significant differences were found in either of these measures in the AST ( $\bar{RR_{x}}$ : z=−1.244 , r=0.415 , p=0.214 ; $\bar{RR_{y}}$ : z=−1.599 , r=0.533 , p=0.110 ). Thus, the AC comparison was significant in both AST and VST, whereas significant differences in $\bar{RR_{x}}$ and $\bar{RR_{y}}$ were observed only in the VST.

## 5 Discussion

### 5.1 Questionnaire results

In the AST, both the sense of agency (SoA) and sense of body ownership (SoO) significantly improve under both the SA and EA conditions. Therefore, the AST helps enhance the subjective sense of body ownership and agency over the avatar. Inamura et al. indicated that the sense of agency is not considerably influenced by avatar size, but the sense of body ownership is. The difference in the sense of body ownership results can be attributed to the difference in the avatar magnification factor. In Inamura et al., the forearm length of the EA was double the original size; in the present study, the EA forearm length is 1.5 times the original size, thus possibly not inducing a significant difference in the sense of body ownership.

Regarding the influence of avatar size on the sense of agency observed in this study, Inamura et al. found a slight decrease in the sense of agency with the double-sized avatar relative to the standard-sized avatar, but the difference was not considerable. This could be related to the avatar display lags; in the current study, this is approximately 20 ms, but it was 200±20 ms in Inamura et al. The threshold for sensing the sense of agency is 230 ms (Shimada et al., 2010), suggesting that Inamura et al. had lower sensitivity to the sense of agency, making their findings less susceptible to the impact of avatar size.

In the VST, only the sense of body ownership under the EA condition improves significantly, with no significant changes observed otherwise. The voluntary periodic movement of the right arm required in the AST may improve the sense of agency. On the contrary, in the VST, the right arm with the altered avatar size is fixed, and no voluntary movement occurs, so the sense of agency does not change.

This phenomenon is particularly noticeable under the SA condition of the VST, where the avatar arm length is similar to the forearm length. Even when the position is slightly misaligned under the EA condition, no physical body exists in the area where the fingertips are touching the avatar. Thus, the skin around the forearm is not stimulated, and the sense of body ownership is not compromised. In this state, the information for recognizing the body comes solely from the visual and fingertip feedback, leading to an improvement in the sense of body ownership. As seen in the effect sizes in Figure 7, the effect size of the sense of body ownership under the EA condition of the VST is almost equal to that of the sense of body ownership under the SA condition of the AST, suggesting that the VST has considerable potential to enhance the sense of body ownership as long as no contact occurs with the participant’s physical body.

### 5.2 Measurement of proprioceptive drift

Significant differences in proprioceptive drift measurements were observed in the VST between the pretest–EA test and SA test–EA test results, indicating that proprioceptive drift occurs in the elongation direction when using the elongated avatar. The same directional trend was observed in the AST but did not reach statistical significance after the Bonferroni correction. For the pretest–SA test comparison, no significant differences are found, with the normalized forearm length remaining at approximately 0.82–0.84, regardless of the type of habituation task or the pre- and post-habituation task conditions. In the EA test, proprioceptive drift occurs, but the magnitude differs between the AST and VST. With the pretest as the baseline, a forearm-length directional proprioceptive drift of approximately 0.04 occurs in the EA test in the AST, whereas approximately 0.08 occurs in the VST, corresponding to increases of approximately 4.9% and 9.6% relative to the pretest values, respectively. Considering that the forearm length of the EA is 1.5 times larger than that of the SA, VST more strongly aligns the perception of body size with the avatar size.

Inamura et al. reported a proprioceptive drift of 1.2±1.6 cm when using an avatar with double the normal forearm length. In our study, the average forearm length of the participants is 44.9±4.75 cm. Therefore, in absolute terms, the proprioceptive drift is 1.8±0.19 cm in the AST and 4.5±0.48 cm in the VST. Despite the higher forearm length multiplier of Inamura et al., the absolute proprioceptive drift observed in our experiment is larger. This can be attributed to the difference in the duration of the habituation tasks. The duration of the habituation task in Inamura et al. (equivalent to the AST) was 60 s, whereas ours is 5 min. The duration of synchronous visuotactile stimulation affects the amount of proprioceptive drift (Rohde et al., 2010); thus, this duration difference can influence the absolute amount of proprioceptive drift.

The difference in proprioceptive drift between the AST and VST may be due to VST, where the length of the right arm is perceived not only visually but also through other body parts interacting with the right arm, thus updating the body schema. In the VST, the forearm angle is fixed at approximately 30°, whereas in the proprioceptive-drift measurement, the forearm angle is fixed at approximately 90°. Despite this posture difference during measurement, proprioceptive drift occurs after VST under the EA condition, suggesting that VST actions may contribute to the general updating of the body schema. The results of the pretest-normalized proprioceptive drift also indicate the magnitude of the proprioceptive drift effect caused by VST.

In the pretest, the normalized forearm length is below 1. This suggests a tendency to underestimate the arm length in situations relying solely on somatosensory input, regardless of avatar experience. Longo and Haggard (2010) and Matsumiya (2022) reported a tendency of participants to underestimate finger lengths when asked to identify each finger’s position. Furthermore, Longo et al. conducted a similar experiment with participants who were congenitally missing one arm and reported phantom limbs, finding that this perceptual distortion was consistent for both the intact arm and the phantom limb (Longo et al., 2012). Based on these findings, Longo et al. argued that the representation of the body structure is independent of visual and somatosensory input, derived from an innate representation of the body in the brain. Avatar habituation can be advanced by understanding the characteristics of this independent body representation (called body form by Proske and Gandevia (2012), distinct from the body schema or body image) and developing avatar habituation methods aligned with it.

### 5.3 Reaching task

In both the AST and VST, significant differences in AC are observed between the SA and EA tests. Considering the direction based on the mean values, AC significantly decreases under both conditions. Thus, the clearance from the obstacle decreases due to avatar elongation.

Regarding $\bar{RR_{x}}$ and $\bar{RR_{y}}$ , significant differences are found only in the VST. The mean values show that $\bar{RR_{x}}$ decreases while $\bar{RR_{y}}$ increases, indicating a shift in the reached endpoint toward the upper left relative to the goal circle. No significant differences are observed in the AST.

In the experimental setup, only the cylindrical obstacles and the body avatar are rendered. Under these conditions, the participants can be assumed to set their avatar, especially the visible forearm, as the reference for the surrounding spatial coordinates. When the forearm length is elongated, the surrounding spatial coordinates may be distorted toward the original forearm length as the reference. Hence, even if the avatar size changes, the participants subjectively perceive their forearm length as constant and reinterpret the spatial scale accordingly. In this scenario, the forearm elongation of the EA leads to the perception of spatial contraction, resulting in reduced clearances. However, if this hypothesis were correct, then $\bar{RR_{y}}$ should decrease; by contrast, it remains unchanged in the AST and increases in the VST. This discrepancy suggests discrepancies in the spatial coordinate perception scales between proximal and distal spaces. In the proximal space, the lack of perspective-induced distortion may allow for an intuitive coordinate system based on forearm length. By contrast, in the distal space, perspective distortions reduce the reliability of the visual forearm length, so an absolute size reference stored in the body schema may guide the reach distance. Therefore, we hypothesize that in the proximal space, participants construct a coordinate system based on the relative sizes of the forearm and surrounding objects, whereas in the distal space, they use a tactile reference of the reach distance. This hypothesis explains the reduced clearance in the proximal space and the unshifted or shifted toward the distal position of endpoints in the distal space. The avatar is displayed during each trial calibration, suggesting that the visual information of forearm length may cause such coordinate distortions.

Additionally, the proprioceptive-drift measurement results indicate greater drift in the VST than in the AST. Therefore, the subjective arm length change in the VST may expand the perceived reachable area, distorting the distal coordinates toward the depth. This contradicts Nakamura et al.’s findings, where the reach distance in a reaching-switch task decreased to the avatar elongation (Nakamura et al., 2014). This divergence may be due to the task-oriented ownership/agency induction produced by elongated avatars in Nakamura et al.’s study, which incorporates motor control adaptation. In the current research, avatar-related ownership and agency are dissociated from the task context, leading to the observed differences. The present study differs from previous studies in that VST also applies feedback to the “not only touched finger but also touching one” and shows the possibility of intervening in motor control strategies (Cheymol et al., 2022).

Body schemas are generated in the parieto-insular vestibular cortex (Lenggenhager and Lopez, 2016), and the related information was used as the initial condition for generating motor programs in the motor loop comprising the basal ganglia and cerebellum (Middleton and Strick, 2000). The change in the motor control strategy may have been induced by the change in the initial conditions for generating movement by updating the body schema with VST.

### 5.4 Comparison of AST and VST

The AST and VST induced proprioceptive drift and reduced obstacle clearance (AC) under the EA condition. This suggests that avatar elongation consistently updates body-size representation and alters proximal motor planning, regardless of the habituation method. These shared effects imply that multisensory congruence between the visual avatar and proprioceptive feedback is sufficient to trigger implicit body schema updating.

However, the two methods differed in the pattern of effects. AST produced broader effects on subjective questionnaire measures, whereas VST showed more limited subjective effects but clearer effects on implicit body-schema- and motor-planning-related measures. AST produced significant improvements in SoO and SoA under SA and EA conditions. This reflects that continuous voluntary arm movement provides strong proprioceptive-motor cues for agency. By contrast, VST selectively improved SoO under the EA condition only. This is likely because the fixed right arm during self-touch precludes the continuous efference-copy signals needed for agency. Despite the narrower effects of the questionnaire, the VST produced a proprioceptive drift similar to that of the AST (0.08 vs. 0.04 normalized units; 4.5 vs. 1.8 cm in absolute terms) and uniquely shifted the reaching endpoint ( $\bar{RR_{x}}$ , $\bar{RR_{y}}$ ), suggesting that tactile-proprioceptive self-contact provides rich multisensory input for updating the body schema than visuomotor synchrony alone.

Given the small sample size (n=10) and the absence of direct between-condition statistical comparisons, these interpretations should be treated as preliminary information. Further research with larger samples and randomized condition order is necessary to confirm and quantify the differential effects of AST and VST on body schema updating.

### 5.5 Limitations and future work

The present study is not without its limitations. First, the small sample size ( n=10 ; n=9 for the reaching task) limited the statistical power and generalizability of the findings. Future studies should include a larger cohort with an *a priori* power analysis. Second, the questionnaire was administered orally, which may have introduced experimenter demand. Future studies should consider the use of a standardized, self-administered questionnaire to mitigate this potential bias. Third, the accuracy of finger-position tracking in the VST was insufficient to guarantee true fingertip-to-fingertip contact. In practice, the fingertip of the left index finger frequently contacted the joints or palm of the right hand rather than the fingertip. This created a discrepancy between visual/haptic feedback and actual somatosensory input, which likely suppressed improvement in subjective experience (SoO), particularly under the SA condition. Future work should use anesthetic sprays and finger cots to isolate fingertip contact. This will validate the assumption that skin contact and colocated vibration have equivalent effects on body schema updating. Furthermore, the VST includes a motor-learning component because participants attempted to touch with their own fingers; therefore, the motor and tactile modalities were not fully separable. The specific contribution of the vibration stimulus should be examined in a condition without vibration. In this experiment, we investigated avatar-induced body-representation changes immediately after each habituation task. In practical applications, such as robot operation or rehabilitation, the ownership/agency and body-schema-related effects should be sustained. The RHI reportedly diminishes approximately 1 min after the cessation of simultaneous visuotactile stimulation (Finotti et al., 2023), so the robustness of VST to this temporal decay should also be investigated. Future work should therefore examine whether long-term exposure to VST sustains changes in body schema and motor control strategies in patients with motor impairment and altered proprioception.

## 6 Conclusion

In this study, an avatar habituation method was designed in a VR space based on the STI. This method was then compared with previous research to understand changes in the body schema induced by avatar experience.

As a preliminary investigation, the initial findings indicate larger alterations in ownership/agency questionnaire measures and proprioceptive drift metrics for the AST compared with previously reported outcomes. This improvement was tentatively attributed to factors such as the reduced latency between movements and the avatar display, as well as the extended duration of our habituation task. These findings suggest that the same habituation task may yield stronger effects related to ownership/agency and proprioceptive drift through enhanced VR system comfort and longer habituation. However, the limits of such improvements require investigation in larger-scale studies.

Preliminary evidence suggests that the proposed VST method improves the SoO under the EA condition. This is supported by a relatively large proprioceptive drift ( 4.5±0.48 cm in absolute terms under EA; cf. 1.2±1.6 cm reported by Inamura et al. with a 2 × avatar), as well as changes in the reach trajectory. These preliminary findings suggest that VST may affect ownership/agency indices and body-schema-related measures, particularly when the avatar has a different body size from the user. The act of voluntarily reaching out and touching oneself on the avatar may facilitate updates in the body schema, although this interpretation would require confirmation through future studies with larger samples. Under the SA condition, there was no improvement in the SoO or agency. The interplay between simultaneous visuotactile stimulation within the VR environment and physical body contact in VST may have hindered the enhancement in the SoO. Therefore, the consistency between visual and tactile information should be enhanced to improve body ownership and agency through VST.

In this experiment, we investigated the extent of ownership/agency and body-schema-related effects immediately after each habituation task. In practical applications, such as robot operation or the use of these techniques for rehabilitation, the ownership/agency and body-schema-related effects should be sustained.

## Data Availability

The raw data supporting the conclusions of this article will be made available by the authors, without undue reservation.
