# Supplementary material for: A methodological study of virtual self-touch: effects on perception and motor control strategies
Source: Front Bioeng Biotechnol. 2026 Jun 22;14:1819228. doi: 10.3389/fbioe.2026.1819228 (PMC13333635; doi:10.3389/fbioe.2026.1819228)
Supplement: Supplementary file 1 [file Supplementaryfile1.pdf]

APPENDIX

Hardware Specifications

The controller unit of the haptic feedback device was constructed around an Arduino Uno microcontroller (Arduino). The vibrators were small eccentric motors (FM34F, Tokyo Parts Industrial). The HMD was a Meta Quest Pro (Meta), which has a field of view of approximately 120 degrees (Meta, 2024b).

Software and Network Configuration

The VR application was developed using Unity (version 2022.3.34f1, Unity Technologies) with the Meta XR Interaction SDK (Meta, 2024c). The control application communicated with the HMD via Wi-Fi using a TL-WR902AC router (TP-Link) on a 5-GHz band with no internet access. Data exchange utilized TCP with a custom protocol. The operator monitored participants' views via USB using Meta Quest Developer Hub (Meta, 2024a). Avatar base meshes were derived from Blender's Human Base Meshes (version 1.1) and exported in VRM format (v0.8) (VRM Consortium, 2024).

Supplementary Statistical Tables

Table S1: Wilcoxon signed-rank test results for questionnaire measures. Pre- vs. post-comparisons for sense of agency (SoA) and body ownership (SoO) under each avatar condition (SA, EA) and habituation method (AST, VST).

Habituation	Measure	Contrast	n	z	r	p	Sig.	CI95 low	CI95 high
AST	SoO	SA	10	-2.121	0.671	0.034	TRUE	-1	0
AST	SoO	EA	10	-2.070	0.655	0.038	TRUE	-1.5	0
AST	SoA	SA	10	-2.646	0.837	0.008	TRUE	-1	0
AST	SoA	EA	10	-2.000	0.632	0.046	TRUE	-1	0
VST	SoO	SA	10	-0.577	0.183	0.564	FALSE	-1	1
VST	SoO	EA	10	-2.111	0.667	0.035	TRUE	-1	0
VST	SoA	SA	10	-1.807	0.571	0.071	FALSE	0	2
VST	SoA	EA	10	-0.378	0.120	0.706	FALSE	-1	1

Table S2: Wilcoxon signed-rank test results for proprioceptive drift (normalized forearm length). Within-group comparisons (Pre/SA/EA, Bonferroni-corrected across three pairs).

Habituation	Contrast	n	z	r	p	p_{adj}	Sig.	CI95 low	CI95 high
AST	Pretest vs. SA Test	10	-0.357	0.113	0.721	1.000	FALSE	-0.040	0.017
AST	Pretest vs. EA Test	10	-1.478	0.467	0.139	0.418	FALSE	-0.088	0.009
AST	SA Test vs. EA Test	10	-1.682	0.532	0.093	0.278	FALSE	-0.061	0.009
VST	Pretest vs. SA Test	10	-0.255	0.081	0.799	1.000	FALSE	-0.043	0.043
VST	Pretest vs. EA Test	10	-2.701	0.854	0.007	0.021	TRUE	-0.118	-0.045
VST	SA Test vs. EA Test	10	-2.803	0.886	0.005	0.013	TRUE	-0.090	-0.050

Table S3: Wilcoxon signed-rank test results for pretest-normalized proprioceptive drift (PD). SA vs. EA comparisons were conducted separately for each habituation method (AST, VST).

Habituation	Contrast	n	z	r	p	Sig.	CI95 low	CI95 high
AST	SA vs. EA	10	-1.682	0.532	0.093	FALSE	-0.061	0.009
VST	SA vs. EA	10	-2.803	0.886	0.005	TRUE	-0.090	-0.050

Table S4: Wilcoxon signed-rank test results for reaching task metrics (SA vs. EA). Average clearance (AC), reaching rate in x ($\overline{RR_x}$), and y ($\overline{RR_y}$) axes for each habituation method (AST, VST).

Measure	Habituation	Contrast	n	z	r	p	Sig.	CI95 low	CI95 high
$\overline{AC}$	AST	SA vs. EA	9	-2.666	0.889	0.008	TRUE	0.004	0.027
$\overline{AC}$	VST	SA vs. EA	9	-2.310	0.770	0.021	TRUE	0.002	0.039
$\overline{RR_x}$	AST	SA vs. EA	9	-1.244	0.415	0.214	FALSE	-0.018	0.030
$\overline{RR_x}$	VST	SA vs. EA	9	-2.073	0.691	0.038	TRUE	-0.010	0.039
$\overline{RR_y}$	AST	SA vs. EA	9	-1.599	0.533	0.110	FALSE	-0.045	0.002
$\overline{RR_y}$	VST	SA vs. EA	9	-2.073	0.691	0.038	TRUE	-0.044	0.002

REFERENCES

[Dataset] Meta (2024a). Meta quest developer hub. https://developer.oculus.com/meta-quest-developer-hub/. Visited on 2024-09-04

[Dataset] Meta (2024b). Meta quest pro tech specs. https://www.meta.com/jp/quest/quest-pro/tech-specs/. Visited on 2024-09-04

[Dataset] Meta (2024c). Meta xr interaction sdk. https://assetstore.unity.com/packages/tools/integration/meta-xr-interaction-sdk-265014. Visited on 2024-09-04

[Dataset] VRM Consortium (2024). VRM 3D humanoid avatar file format for VR. https://vrm.dev/en/. Visited on 2024-09-04
